# The Role of Selenium in Atherosclerosis Development, Progression, Prevention and Treatment

**DOI:** 10.3390/biomedicines11072010

**Published:** 2023-07-17

**Authors:** Siarhei A. Dabravolski, Vasily N. Sukhorukov, Alexandra A. Melnichenko, Victoria A. Khotina, Alexander N. Orekhov

**Affiliations:** 1 Department of Biotechnology Engineering, Braude Academic College of Engineering, Snunit 51, P.O. Box 78, Karmiel 2161002, Israel; 2 Institute of General Pathology and Pathophysiology, 8 Baltiyskaya Street, Moscow 125315, Russia; vnsukhorukov@gmail.com (V.N.S.); sasha.melnichenko@gmail.com (A.A.M.); nafany905@gmail.com (V.A.K.); a.h.opexob@gmail.com (A.N.O.)

**Keywords:** selenium, cardiovascular, selenoproteins, atherosclerosis, endothelial dysfunction

## Abstract

Selenium is an essential trace element that is essential for various metabolic processes, protection from oxidative stress and proper functioning of the cardiovascular system. Se deficiency has long been associated with multiple cardiovascular diseases, including endemic Keshan’s disease, common heart failure, coronary heart disease, myocardial infarction and atherosclerosis. Through selenoenzymes and selenoproteins, Se is involved in numerous crucial processes, such as redox homeostasis regulation, oxidative stress, calcium flux and thyroid hormone metabolism; an unbalanced Se supply may disrupt these processes. In this review, we focus on the importance of Se in cardiovascular health and provide updated information on the role of Se in specific processes involved in the development and pathogenesis of atherosclerosis (oxidative stress, inflammation, endothelial dysfunction, vascular calcification and vascular cell apoptosis). We also discuss recent randomised trials investigating Se supplementation as a potential therapeutic and preventive agent for atherosclerosis treatment.

## 1. Introduction

Selenium (Se) is an essential micronutrient incorporated into selenoproteins as the amino acid selenocysteine (Sec) and involved in metabolic, cardiovascular, thyroid and immune system functioning. In food (including animal and plant products), selenium can be found in organic (γ-glutamyl methylselenocysteine, selenomethionine, methylselenocysteine and selenocysteine) and inorganic forms (sodium selenate and sodium selenite) [1]. The biosynthesis of Sec is unique because it occurs on the UGA codon, which is the stop codon (opal). The canonical mechanism for Sec incorporation suggests that the Selenocysteine Insertion Sequence (SECIS) stem-loop structure in all 25 mammalian selenoprotein mRNAs’ 3′ UTR is the major RNA element controlling Sec insertion. First, tRNA (tRNA^(Ser)Sec^) is aminoacylated with Serine, and then it is phosphorylated to form phosphoseryl-tRNA^(Ser)Sec^. Then, Sec-charged tRNA is produced by a transfer of a Se group from selenophosphate [2]. Recently definitions of the Selenoprotein S (SelS) mechanism suggest that the correct UGA recoding and incorporation of Sec instead of the stop codon involves a proximal stem loop (PSL) and a conservative SelS Positive UGA Recoding (SPUR) element on the first 91 nucleotides of the 3′ UTR (untranslated region) of the SelS [3].

Reactive oxygen species (ROS) are crucial for normal mitochondrial function, disulphide-bond formation, protein folding and cellular signalling. However, surplus ROS causes protein and DNA damage, promotes inflammation and cell death by ferroptosis and/or apoptosis and disturbs normal cellular proliferation, thus contributing to the development of atherothrombotic states [4,5]. The lack of oxidants, however, interrupts normal cellular responses, promoting insulin resistance [6,7,8]. Selenoproteins play important roles in antioxidant and redox biology (methionine-sulfoxide reductase (MsrB1), thioredoxin reductases (Txnr) and glutathione peroxidases (GPX)) and modulate ROS levels (Selenoprotein T, Selenoprotein S and Selenoprotein P) [9]. Low Se supply disrupts normal synthesis of stress-induced selenoproteins, which subsequently increases oxidative stress and inflammation, affecting cardiovascular health [10]. 

The role of selenoproteins and Se supplementation in the development of various cardiovascular diseases has been reviewed previously [11,12]. Atherosclerosis is a chronic inflammatory disease, which is characterised by a deposition of modified lipids within the arterial wall, immune cell infiltration, lesion formation and subsequent plaque development [13,14]. Inflammation and oxidative stress are considered as the main factors promoting atherosclerosis initiation and progression [15,16]. Nowadays, advanced atherosclerosis-associated cardiovascular diseases (such as coronary heart disease, ischaemic stroke or peripheral artery disease) are the leading mortality cause, with 17.9 million deaths (or 32% of all deaths per year) worldwide [17]. In this focused review, we aim to review recent studies on Se metabolism, highlighting the molecular mechanisms associated with antioxidant and redox activities in the context of its involvement in the development and pathogenesis of atherosclerosis. In addition, we discuss Se supplementation as a potential therapeutic and preventive agent for atherosclerosis treatment. For this purpose, 88 recent (the past 3–5 years) papers were searched and selected from the NLM (Pubmed and Medline) database in April–May 2023. 

## 2. Molecular Basis of the Se-Mediated Athero-Protective Activities

**Figure 1:**
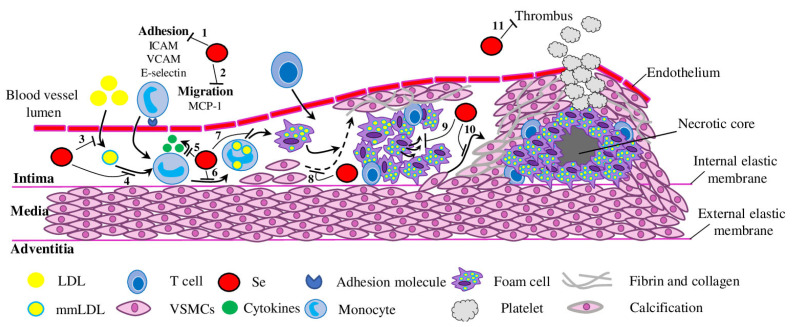
Mechanisms for the Se-mediated prevention of atherosclerosis. Se and selenoproteins may interfere in atherosclerosis development and progression by inhibiting adhesion (1) and migration (2) of monocytes, LDL modification (3) and accumulation (4), cytokine secretion (5), mmLDL uptake (6) and foam cell formation (7), VSMC migration and atherosclerotic lesion formation (8), apoptosis (9), calcification (10) and thrombosis (11). Low-density lipoprotein—LDL; mmLDL—multiple modified low-density lipoprotein; VSMCs—vascular smooth muscle cells.

Over the last decade, many studies have explored the molecular and cellular mechanisms by which Se and selenoproteins prevent atherosclerosis development and progression [18]. The results of these studies demonstrated that selenoproteins may affect the key processes in atherosclerosis development, including ROS-induced oxidative stress, inflammation (eicosanoid metabolism, immune cell adhesion and migration, and foam cell formation), vascular calcification, vascular cell apoptosis and endothelial dysfunction (normalising of NO levels) (Figure 1). Further, we discuss in detail the recent findings deciphering the role of selenoproteins in these processes. 

### 2.1. Oxidative Stress and Se

The insufficient antioxidant production and rise of oxidants (such as reactive oxygen and nitrogen species (RONS)) lead to a potentially damaging process termed oxidative stress. Data from multiple laboratory and clinical experiments have proven that oxidative stress plays a crucial role in atherosclerosis initiation and pathogenesis. Various radicals and oxidants (such as singlet oxygen, hypochlorous acid, peroxynitrite, thiyl radical and others) generate dangerous oxidation products (such as oxysterols, nitrated and chlorinated products, hydroxylinoleate isomers and others), thus contributing to atherosclerosis development [19]. For several decades, a primary role in atherosclerosis initiation was suggested for oxidised lipids, in particular, oxidised low-density lipoproteins (oxLDL). However, the causative role of oxLDL in atherosclerosis initiation was not supported by multiple experimental and clinical data [20,21], providing, nevertheless, evidence for other types of lipid modifications (such as desialylated LDL) for therapeutic intervention and proposing the concept of multiple modified LDL (mmLDL) [13,22].

Results of many experiments support the idea that anti-oxidant properties are mainly responsible for Se-mediated anti-atherosclerotic effects. Se supplementation-mediated anti-oxidant properties have been proven in various cell cultures in in vitro and in vivo models. For example, Se supplementation in the form of sodium selenite or selenomethionine (SeMet) enhanced expression of *Txnr* and *GPX* in placental trophoblast cell lines (Swan-71, JEG-3 and BeWo) and reduced ROS levels in a dose-dependent way [23]. The protective effect of Se (sodium selenite) was also demonstrated on GPX-1-overexpressing or knockdown lymphocytes treated with cell damaging toxic agent Deoxynivalenol (DON). While DON can cause oxidative damage in both GPX-1 lines (overexpressing and knockdown), Se supplementation can antagonise DON’s toxic effect in a GPX-1–dependent way [24]. Similarly, experiments on rat cardiac myoblasts treated with Se demonstrated improved *Txnr* and *GPX* expression and superoxide dismutase (SOD) activity. In addition, Se (sodium selenite) treatment ameliorated H_2_O_2_-induced cell apoptosis and reduced *malondialdehyde* (MDA) concentration [25].

In vivo experiments have supported the beneficial effect of Se supplementation on the redox status of the cardiovascular system. Feeding of the spontaneously hypertensive rats (SHR) with Se (in the form of SeMet) significantly increased whole blood and aortal GPX-1 activity. In addition, the expression of *endothelial NOS* and lipid peroxidation levels in the aortic wall and serum levels of antibodies against advanced glycation end-products was decreased [26]. Sodium selenite supplementation alleviated oxidative damage induced by sodium azide (NaN_3_) in mice. In particular, levels of cardiac lipid peroxidation were decreased, and activities of CAT, SOD, glutathione reductase and GPX as well as total antioxidant capacity (TAC) levels were increased after Se administration [27]. Recently, athero-protective properties of a combined Se (in the form of sodium selenite)/Vitamin E administration were studied in rats treated with the toxic pesticide Bifenthrin. Bifenthrin treatment increased the levels of total cholesterol, LDL cholesterol, oxLDL and native LDL-apoB-100 in both plasma and aorta. In addition, plasma levels of pro-inflammatory cytokines (IL-2, IL-6 and TNF-α) and arterial expression of scavenger receptors (*CD36*) and LDL receptors (LDLRs) were increased due to Bifenthrin toxicity. However, administration of combined antioxidants (Se/Vitamin E) ameliorated alterations in the lipid profile in the aorta and plasma and prevented the pro-antherogenic effect of Bifenthrin treatment in rats [28]. 

Overall, Se supplementation improves antioxidant status and reduces oxidative stress-induced damage to biomolecules, cells, organs and tissues, and thus is protective against atherosclerotic events. The beneficial effects of Se supplementation in combination with other antioxidants have been confirmed in both in in vitro and in vivo models, suggesting the presence of a synergistic effect between Se and other antioxidants.

### 2.2. Inflammation and Se

#### 2.2.1. Se and Eicosanoid Metabolism

Eicosanoids are synthesised from polyunsaturated long fatty acid chains derived from ω-3 (n-3) and ω-6 (n-6) fatty acids. As a group of lipids mediators, they represent the oxidized lipid products and have been shown to mediate receptors involved in Ca^2+^ influx and transcription factors that potentiate the propagation of an acute immune response [29]. The specific eicosanoids associated with the pathogenesis of atherosclerosis have been found to derive mostly from myeloid-derived granulocytes as well as epithelial and endothelial cells (ECs). There are three main pathways recognised in eicosanoid biosynthesis, defined by the main enzyme involved: the cyclooxygenase (COX), lipoxygenases (LOX) and cytochrome P450 (cyP450) pathways [30]. The association between eicosanoids and cardiovascular systems has been long known, and in normal conditions, ECs produce several eicosanoids to regulate vascular tone and homeostasis and prevent platelet aggregation [31]. 

GPX-1 and GPX-4 metabolise hydroperoxides produced by LOX and COX eicosanoid biosynthesis pathways. Se deficiency, subsequently, decreases *GPX-1* and *GPX-4* activity and expression, which leads to hydroperoxide accumulation and imbalance between synthesized eicosanoids, most notably, pro-inflammatory (prostaglandin E_2_ and thromboxane A_2_) and anti-inflammatory (prostaglandin D_2_, cyclopentenone PG and Δ^12^ prostaglandin J_2_) prostaglandins [32]. Such a mechanism was reported in macrophages and termed “eicosanoid class switching”; it is highly controlled by selenoproteins [33]. As it was recently shown on LPS (lipopolysaccharide)-stimulated macrophages under normal conditions, RONS activated the COX-1-dependent pathway to produce anti-inflammatory enzymes. However, under inflammation conditions, surplus RONS activated the COX-2 pathway, which produced pro-inflammatory enzymes. Application of Se-rich maize extracts down-regulated pro-inflammatory *COX-2* and *microsomal prostaglandin E_2_ synthase-1* genes and up-regulated *GPX-1* and *haematopoietic PGD_2_ synthase* [34]. 

Recent experiments on THP-1 monocytes confirmed the important role of Se level in NF-κB-dependent inflammatory signalling and biosynthesis of inflammatory lipid mediators in monocytes differentiated into macrophages. Se supplementation increased the expression of *selenoproteins H* and *F*, and differentiation further enhanced selenoprotein F while decreasing *selenoprotein H* expression. Most importantly, Se facilitates the expression of LPS-induced NF-κB target genes (*COX-2* and *TNF-α*) and the release of COX- and LOX-derived lipid mediators 12- and 15- hydroxyeicosatetraenoic acids, thromboxanes B_2_ and B_3_, and arachidonic acid, docosahexaenoic acid and eicosapentaenoic acid [35]. 

20-hydroxyeicosatetraenoic acid (20-HETE), a metabolite of arachidonic acid produced by the cyP450 pathway, is linked to oxidative stress during severe inflammation and associated with vascular dysfunction and tissue damage [36]. Experiments on bovine aorta primary endothelial cells showed that 20-HETE decreased endothelial barrier integrity and total glutathione content and increased RONS production. The antioxidant Vitamin E alone did not prevent complete loss of barrier integrity, while cells supplemented with selenium (sodium selenite) were resistant to 20-HETE–induced decreases in barrier integrity [37]. 

Collectively, these results indicate that Se supplementation could potentially diminish the biosynthesis of pro-inflammatory eicosanoids and cytokines during atherosclerosis. The current knowledge suggests that the antioxidant and anti-inflammatory properties of Se are promotive and interdependent. However, the role of Se in the modulation of redox-dependent signalling and downstream lipid mediator profiles in macrophages requires further investigation. 

#### 2.2.2. Se and Leukocyte Recruitment and Migration

During the atherosclerotic lesion initiation, the pro-inflammatory state of the ECs recruits monocytes and promotes their migration across the endothelium. The inflammatory cytokines (such as TNF-α or interleukins) induce the expression of adhesion molecules by the ECs (such as *P-* and *E-selectins*, *intercellular adhesion molecule-1* (*ICAM-1*) and *vascular cell adhesion molecule-1* (*VCAM-1*)). Additionally, several hydroperoxides (such as fatty acid hydroperoxides or H_2_O_2_) can induce the expression of cell adhesion molecules and promote atherosclerosis development [38]. 

The connection between Se status and the expression of adhesion molecules has been long known. Early experiments proved that Se (sodium selenite) inhibited the TNF-α–mediated increase in ICAM-1, VCAM-1 and E-selectin levels in a dose-dependent manner [39]. Furthermore, Se supplementation (in the form of SeMet) reduced atherosclerotic plaque formation, stabilised lesions and improved vessel function in Apolipoprotein E–deficient (ApoE^−/−^) mice fed a high-fat diet. Importantly, the lesion accumulation of M1 inflammatory-type macrophages and extracellular trap formation (as a result of neutrophils cell death—NETosis) were decreased after SeMet administration [40]. On the other side, Se deficiency was associated with reduced GPX-1 activity, enhanced expression of *ICAM-1* and *E-selectin*, and cytokine (*IL-1* and *TNF-α*) and H_2_O_2_-mediated neutrophil adhesion to endothelial cells [41]. 

Among other proteins, the antioxidant and redox-regulating properties of GPX-1 are the most studied in the connection to inflammation and expression of adhesion molecules. GPX-1 deficiency enhanced ROS generation accumulation and ICAM-1, MCP-1 (monocyte chemoattractant protein-1) and *VCAM-1* expression, while *GPX-1* overexpression had the opposite effect [42,43]. Recent results showed that GPX-1 deficiency activated MAPK (extracellular-regulated kinase (ERK), p38, c-JUN N-terminal kinase (JNK) and NF-kB pathways and increased *VCAM-1* expression and leukocyte adhesion to the vascular endothelium [44].

A recent investigation of the selenium status in children with systemic inflammatory response syndrome suggested that Se plays a critical role in regulating the magnitude of endothelial activation and the severity of multiple organ dysfunction. Both the low serum selenium and highly reduced fraction of glutathione/total glutathione (GSH/tGSH) correlated with the development of multiple organ failure in children [45]. Further work showed that patients’ clinical severity was associated with decreased plasma Se levels. At the same time, erythrocyte Se concentrations serve as a more reliable Se status biomarker, not affected by the magnitude of the inflammatory response, while associated with selenoprotein P concentrations [46]. Furthermore, erythrocyte Se level was associated with soluble platelet selectin but not with VCAM-1 and ICAM-1 levels. At the same time, plasma Se did not correlate to levels of the adhesion molecules [47].

In total, these results suggest a critical role for Se in inhibiting the expression of adhesion molecules in endothelial activation, having a protective effect on inflammation severity and progression of atherosclerosis and other inflammatory diseases. In comparison to serum Se, erythrocyte Se level serves as a better biomarker of the organismal Se status. 

#### 2.2.3. Reduction of Foam Cell Formation by Se

Accumulation of mmLDL by macrophages leads to the formation of “foam cells”, which are considered as a hallmark of atherosclerosis progression. Some research evidence has suggested that Se may significantly affect macrophage foam cell formation. A high level of GPXs (GPX-1, GPX-3 and GPX-4) was associated with better outcome of acute coronary syndrome patients, suggesting their up-regulation in response to higher oxidative stress [48]. Furthermore, an athero-protective role was suggested for GPX-1. GPX-1 deficiency increased oxLDL-induced foam cell formation and lesion cellularity and accelerated atherosclerosis in GPX1^−/−^ApoE^−/−^ mice through the p44/42 MAPK (p44/42 mitogen-activated protein kinase) pathway. These results were reversed by application of the GPX-mimicking agent ebselen [49]. Similarly, application of D-ribose-L-cysteine, a cysteine-delivery agent, known to increase GPX activity and decrease LDL, lipoprotein(a) and apoB concentrations [50], demonstrated anti-atherosclerotic properties in ApoE^−/−^ mice. In particular, ribose-cysteine treatment increased GSH levels and GPX activity in both liver and erythrocytes, decreased total cholesterol and LDL, and reduced the atherosclerotic lesion area in the aortic sinus and brachiocephalic branch [51]. Such athero-protective effect was also confirmed for another GPX-mimicking organoselenium compound, HBD, in in vitro experiments on murine macrophages. HBD application protected isolated LDL from Cu^2+^-induced oxidation and decreased the foam cell formation in macrophage culture exposed to oxLDL [52]. 

In conclusion, Se supplementation provides athero-protective effects by inhibiting the lipid and protein oxidation and reducing the cellular LDL uptake and, consequently, foam cell formation. At least partially, these effects depend on GPX activity. These results suggest that Se and organoselenium agents are promising pharmacological tools to prevent and interfere with atherogenic processes.

## 3. Se and Vascular Calcification

**Figure 2:**
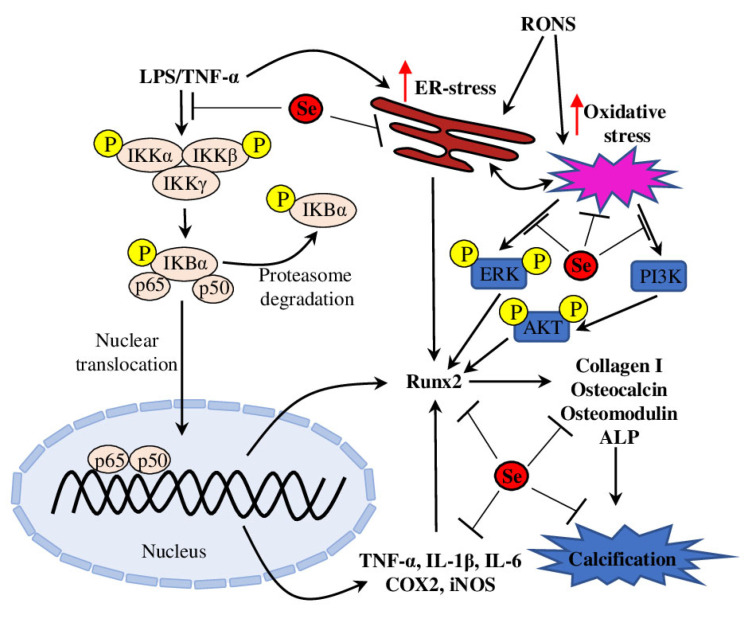
Schematic representation of the proposed inhibitory mechanism of Se in VSMC calcification. Se inhibits activation of the LPS/TNF-α–induced NF-κB signalling pathways and, subsequently, the production of pro-inflammatory molecules and Runx2 activation. Se attenuates ER stress– and oxidative stress–mediated increase of Runx2 (through ERK, PI3K and AKT pathways). Finally, Se directly reduces expression of *Runx2*, *Collagen I*, *Osteocalcin*, *Osteomodulin* and *ALP*, thus inhibiting VSMC calcification. Red arrows represent the increased rate of ER and oxidative stresses.

Vascular calcification is an active, tightly orchestrated process similar to osteogenesis and is associated with adverse effects on plaque stability and vasomotion and, subsequently, can lead to substantial cardiovascular morbidity [53]. Vascular smooth muscle cells (VSMCs) play an integral role in vascular calcification by expressing many bone formation-associated genes (such as Osteoblast-Specific Transcription Factor 2 (Runx2), type I collagen, osteocalcin, alkaline phosphatase and osteomodulin) and miRNAs, which initiate and promote calcium phosphate deposition in the extracellular matrix (Figure 2) [54] as reviewed in [55,56].

Increasing evidence suggests that oxidative stress plays an important role in the pathogenesis of vascular calcification and promotes osteoblastic differentiation of VSMCs. Taking into account the antioxidant properties of Se, it was suggested that Se may be involved in regulating vascular calcification. Further experiments on oxidative stress–enhanced VSMCs supplemented with sodium selenite demonstrated that Se inhibited oxidative stress–activated endoplasmic reticulum (ER) stress and increased GPX content and activity, thus decreasing apoptosis and suppressing oxidative stress. In addition, the activation of phosphatidylinositol 3-kinase/Serine/Threonine Kinase 1 (PI3K/AKT) and extracellular-signal-regulated kinase (ERK) pathways was attenuated, which resulted in decreased osteoblastic differentiation of VSMCs [57].

Recent experiments on LPS and TNF-α–induced VSMCs suggested involvement of the ER localised Selenoprotein S (SelS) in inflammation-induced vascular calcification and osteoblastic differentiation of VSMCs. LPS/TNF-α treatment activated the NF-κB signalling pathway, enhanced *IL-6* expression and ER stress, and increased protein and mRNA levels of Runx2 and type I collagen, alkaline phosphatase activity and calcium deposition content, thus promoting osteoblastic differentiation and VSMC calcification. These effects were aggravated in SelS knockdown VSMC lines. These data suggest that SelS acts as a suppressor of ER stress and NF-κB signalling pathway activation and thus may be beneficial in the prevention of vascular calcification in atherosclerosis [58]. Another molecular mechanism connecting Se status and vascular calcification was defined in experiments on high glucose–induced mouse aortic VSMCs treated with Se-rich *Spirulina platensis* extract. The Se-containing extract inhibited ROS production, reduced ROS-mediated DNA damage and attenuated dysfunction of MAPK and PI3K/AKT pathways, thus effectively inhibiting high glucose–induced calcification [59].

Taken together, Se administration protects vascular cells from inflammation-, ER- and oxidative stress–induced calcification (Figure 2). These beneficial effects are mediated through selenoenzymes (such as GPX) and ER-resident selenoproteins (such as SelS), which act on several known pathways (such as PI3K/AKT, NF-κB and MAPK/ERK). These results provide important mechanistic insights into the potential clinical application of Se-based drugs for treatment and prevention of vascular calcification. 

## 4. Se and Vascular Cell Apoptosis and Autophagy

### 4.1. Anti-Apoptotic Properties of Se

Two intra-cellular stresses (oxidative and ER) can induce apoptosis (a type of programmed cell death) of vascular cells and thus promote atherosclerosis progression [60]. Therefore, Se could protect VSMCs from apoptosis through the inhibition of oxidative and ER stresses. As was shown, rat thoracic aorta VSMCs with silenced *SelS* genes were highly sensitive to peroxide-induced oxidative and ER stresses, higher phosphorylation levels of MAPK and JNK, and higher apoptosis rate compared to control cells [61]. Similar results were obtained on GPX-1 knockout murine embryonic fibroblast cell lines treated with the cardiotoxic chemotherapeutic agent doxorubicin, which causes cardiac apoptosis [62]. Application of doxorubicin increased ROS production and apoptosis rate in mutant cell lines, while Se supplementation (sodium selenite) prevented ROS increase and reduced the level of apoptosis [63]. 

The anti-apoptotic properties of Se supplementation also have been confirmed in in vivo studies. As was demonstrated in the rat model of ischaemia-reperfusion myocardial injury, Se supplementation (in a form of Se-rich polysaccharide extracted from *Aloe vera*) reduced the infarct sizes, increased antioxidant levels (SOD, GSH and CAT) and decreased MDA levels, thus attenuating myocardial damage. In addition, the levels of cardiomyocytic apoptosis, myocardial creatine kinase and lactate dehydrogenase as well as activities of Na^+^-K^+^-ATPase and Ca^2+^-Mg^2+^-ATPase were decreased by Se supplementation. These results suggest that Se-rich plant-derived polysaccharide provides a cardioprotective effect against myocardial injury by modulating endogenous antioxidant levels and protecting rat hearts from ROS-induced myocardial apoptosis [64]. A similar effect was shown on LPS-induced myocardial injury in mice, where Se (sodium selenite) pretreatment decreased oxidative stress, reduced expression of pro-inflammatory cytokines (*IL-1β*, *IL-6* and *TNF-α*) and inhibited myocardial apoptosis (assessed via caspase-3, caspase-8 and caspase-9 activities). Se supplementation also inactivated the Sting pathway (stimulator of interferon genes) [65], which could promote transcription of interferon regulatory factor 3 (IRF3), activate NF-κB and the NLRP3 inflammasome, and induce ER stress, inflammation and apoptosis [66].

Anti-apoptotic properties have also been demonstrated for SelS. Therefore, *SelS* overexpression protected HUVECs from high-glucose-induced apoptosis by reducing cleaved caspase 3 level and by reducing protein kinase CβII, JNK, and B-cell lymphoma/leukemia-2 (Bcl-2) phosphorylation. In contrast, SelS suppression showed the opposite effect, thus suggesting SelS as a promising target for prevention and treatment of diabetic vascular complication [67]. 

### 4.2. Se in Regulation of Apoptosis/Autophagy Balance

An interesting association has been found between Se deficiency and apoptosis/autophagy balance. Autophagy is a special self-cannibalisation mechanism, activated by hypoxia, damaged organelles and nutrient deprivation and starvation, mediated by various proteins (such as Becline-1, microtubule-associated protein light chain 3 (LC3) and ATGs (autophagy-associated genes)), and implicated in various diseases (many types of cancer, neurodegenerative disorders and certain myopathies) [68]. Se deficiency up-regulated mRNA levels of pro-apoptotic proteins (such as Caspase 3, Caspase 8, Caspase 9, Bcl-2 and Bcl-2-associated X Protein (Bax)), while autophagy-associated mRNA (such as Becline-1, Dynein, Mammalian target of rapamycin (mTOR), ATG5 and LC3-1) were down-regulated in chicken cardiomyocytes. These data suggested that under Se deficiency conditions, apoptosis and autophagy may function contradictorily, with activation of apoptosis rather than autophagy as a more pro-survival strategy [69]. 

MicroRNAs (miRNAs) are short non-coding RNAs that are known to regulate gene expression post-transcriptionally and are involved in the organ’s development and various cardiovascular diseases’ pathogenesis [70]. Recently, a crucial role for miRNA was also defined in the Se–autophagy axis. For example, the analysis of the miRNAome of the Se-deficient chicken myocardium showed increased expression of *miR-2954* and *PI3K* as its target gene. Moreover, *miR-2954* overexpression both in vivo and in vitro led to apoptosis and autophagy of myocardial cells during cardiac injury through regulation of the mTOR (Mammalian Target Of Rapamycin) and PI3K pathways [71]. Furthermore, Se deficiency was associated with up-regulated expression of *miR-200a-5p* and induction of necroptosis, a type of unprogrammed cell death (or inflammatory cell death). The target of miR-200a-5p was identified in vivo and in vitro as the ring finger protein 11 (RNF11), which regulates necroptosis in a MAPK- and Receptor-Interacting Serine-Threonine Kinase 3 (RIP3)-dependent way. Indeed, miR-200a-5p knockdown cardiomyocytes were resistant to standard necroptosis triggers (z-VAD-fmk) and showed enhanced cell survival against necrosis [72]. Considering the close association between RIP3 and myocardial fibrosis and autophagy [73], these results present a novel regulatory model of myocardial necrosis in heart disease. 

Recently, the effect of Se supplementation on heart failure development was demonstrated. In particular, Se application improved heart antioxidant levels (SOD, SOD2, GPX and GSH), reduced expression of *collagen I* and *III*, *GSK-3β*, *AKT* and *α-SMA*, and reduced the apoptosis rate and ROS levels in TGF-β1–treated rat myoblasts. Additionally, Se treatment up-regulated the level of Sirtuin 1 [74], which promotes autophagy and inhibits apoptosis, thus providing cardioprotective effects in different cardiovascular diseases [75]. In total, these results demonstrate that Se’s cardioprotective effects on heart fibrosis, hypertrophy and failure are mediated through regulating ROS status, apoptosis/autophagy rate, AKT/GSK-3β and Sirt1 pathways. 

## 5. Se and Endothelial Dysfunction

Blood vessel endothelial cells play an important role in regulating vascular homeostasis and exchange between the bloodstream and the surrounding tissues. Nitric oxide (NO), generated from arginine by endothelial nitric oxide synthase (eNOS), is a powerful endogenous vasodilator, which also inhibits cytokine-mediated expression of adhesion molecules and attenuates platelet aggregation and VSMC growth, thus providing anti-atherogenic and anti-thrombotic effects [76]. Oxidative stress, on the contrary, can antagonise these protective effects and promote endothelial dysfunction, which is characterised by a decreased NO bioavailability and normal vasorelaxation responses, impaired barrier function and increased leukocyte adhesion and migration. Furthermore, NO can interact with superoxide and produce highly reactive peroxynitrite, which further enhances oxidative damage, affects DNA, protein and lipid functionality, and causes other cytotoxic effects [77]. As was shown on mice aorta under conditions of hyperglycaemia-induced acute oxidative stress, selenium-containing sugar 1,4-Anhydro-4-seleno-D-talitol (SeTal) decreased superoxide levels and increased basal NO. availability, thus preventing high-glucose-induced endothelial dysfunction and oxidative stress [78]. 

Experiments on primary aortic endothelial cells isolated from GPX1-knockout mice demonstrated reduced Akt phosphorylation and NO bioavailability upon TNF-α treatment, while IκB degradation and TNF-α stimulated phosphorylation of p38, ERK and JNK were prolonged, thus suggesting enhanced NF-κB activity and vascular inflammation. *VCAM-1* expression was also increased in mutant cell lines and aortas. However, those effects were reduced by treatment of GPX-1 mimetic ebselen, which confirms the crucial role of GPX-1–mediated antioxidant defence in the regulation of vascular inflammation and endothelial dysfunction in cardiovascular diseases [44]. Similar results have also been obtained for SelS on TNF-α–treated human umbilical vein endothelial cells (HUVECs). In particular, SelS alleviated the TNF-α–mediated rise of ROS and endothelin-1 levels and enhanced eNOS and NO levels. *SelS* overexpression also prevented other effects of TNF-α treatment: reduced *ICAM-1* and *VCAM-1* expression, inhibited adhesion of THP-1 monocytes to HUVECs, reduced expression of inflammation-related factors (such as *IL-1β*, *IL-6*, *IL-8* and *MCP-1*) and activation of MAPK and NF-κB pathways [79]. 

Recently, the role of Se supplementation in homocysteine-induced endothelial dysfunction was established. Se inhibited apoptosis in homocysteine-treated HUVECs by down-regulating *Caspase-3* and *Bax* expression and by promoting *Bcl-2* expression. The expression and phosphorylation of *eNOS* and *AKT* were increased after Se treatment in a dose-dependent way. However, the beneficial effects of Se treatment were reversed by application of AKT inhibitor (SH-5) [80]. Other research used microfluidic chips to simulate the diabetic vascular endothelial microenvironment and demonstrated that SelS protects human aortic endothelial cells (HAECs) from oxidative stress injury. *SelS* overexpression decreased levels of ROS and endothelin-1 and increased expression of *SOD1* and *SOD2*. Furthermore, *SelS* overexpression increased the p-eNOS/NOS and p-Akt/Akt ratios and total PI3K, but decreased the p-PKCα/PKCα ratio, suggesting indirect activation of the PI3K/Akt/eNOS signalling pathway [81]. Similarly, *SelS* overexpression could protect HAECs from high-glucose/high-oxLDL–dependent endothelial injury and autophagy in an Akt/mTOR-mediated way. Accordingly, *SelS* knockdown caused the opposite effect on HAEC viability [82]. Taken together, these studies suggest that selenoenzymes and selenoproteins play an essential role in preserving normal endothelial function and NO bioavailability.

## 6. Human Studies of Se Supplementation to Treat Cardiovascular Diseases

The association between Se and the cardiovascular system has been known since the 1970s, when Keshan disease, a congestive cardiomyopathy, was described in Se-deficient areas of China and was completely prevented by sodium selenite supplementation [83]. During the past decades, the topic of Se and CVDs has attracted much attention and has been extensively reviewed [11,84,85]. Current knowledge suggests that high body Se is associated with reduced risk of CVD incidence and mortality, while Se deficiency or low Se status are considered as a risk factor for CVD development [86]. Moreover, various clinical studies and randomised trials have investigated the efficiency of Se supplementation to prevent CVDs. Interestingly, these studies have been thoroughly analysed in several meta-analysis papers, in general confirming the beneficial effects of Se supplementation, mostly on secondary parameters (such as levels of C-reactive protein or GPX), but without a direct effect on CVD incidence, coronary heart disease lipid profile or mortality [87,88,89]. The decreased risk of all-cause mortality and CVD was found for supplements where Se was added to antioxidant mixtures, while antioxidant mixtures alone had no such effects [90]. However, Se dose should be carefully calculated, because high Se supply was shown to cause endothelial dysfunction through activation of ER stress and increased ROS production [91]. Further in this section, we focus on studies conducted recently and not covered in the cited meta-analysis papers. 

### 6.1. Human Studies with Se+Coenzyme Q10 Supplementation

During the last decade, the combination of Se (Se-enriched yeast 200 µg/day) and coenzyme Q10 (200 mg/day) supplementation [92] to improve cardiovascular health was thoroughly investigated in a long-term study with elderly Swedish citizens. Over 5 years, the reduction of cardiovascular mortality was accompanied by an improvement of N-terminal-pro hormone BNP (NT-proBNP) (a heart failure marker) levels and on echocardiography [93], C-reactive protein and sP-selectin levels [94], myocardial function and reduced fibrosis through the reduction of inflammation and oxidative stress [95]. Interestingly, the reduced cardiovascular mortality was observed even at 10 [96] and 12 years [97] follow-up. Positive effects were also observed for the plasma levels of von Willebrand factor (vWf) and the plasminogen activator inhibitor-1 (PAI-1) [98], which are markers of coronary endothelial and ventricular dysfunction and myocardial infarction [99,100]. 

Recently, to confirm those results, several validation studies have been conducted. Under the same experimental set-up with elderly male participants, after 18 months of SeQ10 intervention, the levels of 19 (out of 95) metabolites from mevalonate, xanthine oxidase, pentose phosphate and beta-oxidation pathways were significantly changed [101]. Similarly, SeQ10 supplementation over a 4-year period reduced shortening of leukocyte telomere length (LTL), a known biomarker of ageing and cardiovascular diseases [102], reduced levels of fructosamine [103], a risk factor of diabetes, cardiovascular complications and mortality [104], and improved cardiovascular mortality and survival rate. These data suggest that SeQ10 supplementation preserves LTL and reduces cardiovascular mortality [105]. 

### 6.2. Human Studies with Se Supplementation in CAD Treatment

On the contrary, several recent short-term Se supplementation studies have provided more controversial results. For example, 160 patients with coronary artery disease (CAD) received 200 mg of Se daily for 60 days. However, no significant difference was observed between Se, cholesterol and myeloperoxidase (one of the ROS-producing enzymes) levels between the intervention and control groups. Some beneficial effect was found on the level of paraoxonase [106], which is known as an anti-inflammatory, anti-oxidative, anti-atherogenic protein [107]. 

Another study investigated the effect of Se supplementation on 145 patients with CAD with/without metabolic syndrome. After 2 months of 200 mg/daily Se supplementation, no effect on plasma Se, SelP protein or SelP mRNA levels were recorded [108]. Similarly, the expression of *cyclooxygenase-2* (*COX-2*) and *matrix metalloproteinase 9* (*MMP-9*), two crucial enzymes involved in atherosclerosis development, was not changed after 60 days of Se intervention in 41 CAD patients [109]. 

### 6.3. Human Studies with Se Supplementation in Atherosclerosis Treatment

Recently, another trial investigated the effect of Se supplementation among 60 atherosclerotic patients. However, after 8 weeks of treatment, the blood biochemical parameters and lipid profile (insulin, fasting blood sugar, triglycerides, high-density lipoprotein cholesterol and total cholesterol) were unaffected by Se treatment. The only parameter with a significant decrease in the Se-administrated group was low-density lipoprotein cholesterol [110]. Another trial investigated the effect of Se supplementation on the expression of pyroptosis-related genes and biomarkers of oxidative stress in 60 patients with atherosclerosis over 8 weeks. As a result of Se administration, the expression of pyroptosis and inflammation-related genes (*Toll-like receptor 4*, *Apoptosis-associated speck-like protein containing a CARD* (or *ASC*), *NLR Family Pyrin Domain Containing 3, and Nuclear Factor NF-Kappa-B P105 Subunit* (*NF-kB1*)) were down-regulated, while GPX levels were increased and MDA decreased, thus improving antioxidant status and reducing inflammation in atherosclerosis patients [111]. 

In summary, the results from several randomised trials have been inconsistent, and the role of Se supplementation in prevention of atherosclerosis and other CVDs is inconclusive. The baseline Se status of the studied population might be at least partially responsible for the lack of consistency in the conducted trials. Therefore, Se administration may be beneficial for patients with low baseline Se status, while the effect in patients with adequate or high Se status may be neutral or even adverse. The time of supplementation is the second crucial parameter. Among the reviewed trials, the most pronounced effect on the major studied parameters (such as cardiovascular mortality and survival) was demonstrated in long-term trials, while short-term trials reported mostly beneficial effects on secondary parameters (such as particular metabolites levels, for example, LDL or MDA). Available data also suggest that Se supplementation is more effective in combination with other antioxidants (such as Q10 or Vitamin E). 

## 7. Conclusions

The results from conducted randomised trials suggest that long-term Se supplementation in combination with other antioxidants is cardio-protective, reducing the risk of different cardiovascular diseases, cardiovascular complications and mortality. At the same time, the findings from short-term Se supplementation trials are inconsistent, and the cardio-protective effect of Se administration is inconclusive, though it can provide a beneficial influence on some metabolic parameters. Furthermore, a great number of available laboratory studies support the idea that balanced Se intake can provide an athero-protective effect by inhibiting oxidative stress, inflammation, vascular cell apoptosis and calcification, and endothelial dysfunction. Mostly, such athero-protective effects are mediated through the action of selenoenzymes and selenoproteins in the arterial wall.

Importantly, it is necessary to consider complex interactions between essential trace elements (such as Se, zinc, copper, iron and manganese) and non-essential metals (such as lead, cadmium, arsenic, and mercury) in organisms [112]. Similar to the well-documented protective role of Se and Zn, exposure to Cd, Pb, As and Hg has been connected to various heart diseases [113,114]. Thus, possible application of trace elements (primarily Se and Zn) as detoxifying agents against heavy metal–mediated toxicity should be based on the precise identification and clarification of the affected biological processes [115].

However, the research area of Se supplementation for CVD treatment and prevention has many challenges. First, the proper markers to determine the optimal Se status should be defined. The Se blood level itself does not always reflect its functional availability, while it is one of the most easy-to access and widely used markers. Alternatively, the Se level in circulating blood erythrocytes and leukocytes, or the plasma level of particular proteins (such as GPX1 or SelP), are suggested. Secondly, the Se bioavailability from different dietary sources should be considered. Taking into account the toxicity of some Se forms and the possible adverse effect of Se overdose, future investigation of the metabolism and distribution of various Se forms in vivo is of great importance. Finally, in the future, more and larger randomised trials of Se supplementation are necessary to confirm the beneficial effect of Se on atherosclerosis in particular and CVD in general. 

## Data Availability

Not applicable.
